# Wie gut sehen unsere ABC-Schützen?

**DOI:** 10.1007/s00347-020-01194-3

**Published:** 2020-08-24

**Authors:** Heike M. Elflein, Roman Pokora, Denis Müller, Alexander K. Schuster, Klaus Jahn, Katharina A. Ponto, Susanne Pitz, Norbert Pfeiffer, Michael S. Urschitz

**Affiliations:** 1 Augenklinik und Poliklinik, Universitätsmedizin der Johannes Gutenberg-Universität Mainz, Langenbeckstr. 1, 55131 Mainz, Deutschland; 2 Abteilung für Pädiatrische Epidemiologie, Institut für Biometrie, Epidemiologie und Informatik, Universitätsmedizin der Johannes Gutenberg-Universität Mainz, Mainz, Deutschland; 3 Abteilung für Öffentlicher Gesundheitsdienst, Hygiene und Infektionsschutz, Ministerium für Soziales, Arbeit, Gesundheit und Demographie des Landes Rheinland-Pfalz, Mainz, Deutschland; 4 Augenklinik, Bürgerhospital und Clementine Kinderhospital, Frankfurt/Main, Deutschland

**Keywords:** Amblyopie, Schuleingangsuntersuchung, Vorsorgeuntersuchungen, Kindesalter, Rheinland-Pfalz, Amblyopia, School entry examination, Pediatric screening examination, Childhood, Rhineland-Palatinate

## Abstract

**Hintergrund:**

Im Rahmen der kinderärztlichen Vorsorgeuntersuchungen U1 bis U9 von der Geburt bis zum Beginn des sechsten Lebensjahres wird unter anderem das Sehvermögen getestet – eine augenärztliche Vorsorgeuntersuchung im Kindesalter gibt es in Deutschland nicht. Diese Studie untersucht, ob eine Teilnahme an der U8 (am Ende des vierten Lebensjahres) und der U9 (zu Beginn des sechsten Lebensjahres) mit Sehschärfeergebnissen assoziiert ist, die im Rahmen der Schuleingangsuntersuchungen (SEU) erhoben werden.

**Methoden:**

Ausgewertet wurden Daten der SEU des Landes Rheinland-Pfalz der Einschulungsjahrgänge 2009/2010 bis 2014/2015. In diesen Jahrgängen wurde die Sehschärfe mittels Rodenstock-Sehtestgerät (E-Haken; Rodenstock Instrumente GmbH, Ottobrunn, Deutschland) und eigener Korrektur geprüft. Festgehalten wurden reduzierte Sehschärfen von <0,7. Der Zusammenhang zwischen der Teilnahme an den Vorsorgeuntersuchungen U8 bzw. U9 und dem Vorliegen einer ein- und beidseitigen Sehschärfe <0,7 bei SEU wurde mithilfe von multipler logistischer Regressionsanalyse untersucht und für wichtige Störgrößen adjustiert.

**Ergebnisse:**

Daten von 189.704 Kindern (91.041 Mädchen und 98.663 Jungen) aus 35 von 36 Landkreisen konnten eingeschlossen werden. Eine Sehschärfe <0,7 wurde bei 8416 Kindern (4,4 %) ermittelt, in beiden Augen bei 4345 (2,3 %) Kindern. Die Teilnahmequote an der U8 bzw. U9 betrug 93,9 % bzw. 93,3 %. Es bestand eine negative Assoziation zwischen der Teilnahme an der U8 bzw. U9 und einem ein- oder beidseitigen SEU-Visus <0,7 (adjustierte OR: 0,68; 95 %-KI: 0,61–0,75; *p* < 0,01; *N* = 124.467/adjustierte OR: 0,57; 95 %-KI: 0,51–0,65; *p* < 0,01; *N* = 121.496).

**Schlussfolgerungen:**

Es zeigt sich ein hoher Anteil an Kindern mit verminderter Sehschärfe bei der Schuleingangsuntersuchung. Kinder, die in der U8 und U9 untersucht worden waren, hatten eine bessere Chance für eine gute Sehschärfe bei der Schuleingangsuntersuchung.

## Abstract (en)

**Background:**

Within the framework of the German pediatric screening examinations, the checks include visual functions. There is no ophthalmological screening examination in childhood in Germany. This study investigated whether participation in the pediatric screening examinations U8 (at the end of the fourth year of life) and U9 (at the beginning of the sixth year of life) is associated with the results of visual acuity, which are compiled at the school entry examinations (SEU).

**Methods:**

This study evaluated data of the SEU for school the enrollment years 2009/2010–2014/2015 of the State of Rhineland-Palatinate. In these years visual acuity was assessed using the Rodenstock visual testing device (E-hooks; Rodenstock intruments GmbH, Ottobrunn, Deutschland) wearing glasses if present. The association between participation in the U8 and U9 screening examinations and the presence of unilateral and bilateral visual acuity <0.7 was investigated using multiple logistic regression adjusted for important disturbance variables.

**Results:**

Data from 189,704 children (91,041 girls, 98,663 boys) from 35 out of 36 districts were included. A visual acuity <0.7 was measured in 8416 (4.4%) children and in both eyes in 4345 (2.3%) children. The participation rates in the U8 and U9 were 93.9% and 93.3%, respectively. There was a negative association between participation in the U8 and U9 and a unilateral or bilateral SEU visual acuity <0.7 (adjusted odds ratio, OR 0.68, 95% confidence interval, CI 0.61–0.75; *p* < 0.01, *N* = 124,467/adjusted OR 0.57, 95% CI 0.51–0.65;* p* < 0.01, *N* = 121,496).

**Conclusion:**

The proportion of children with visual acuity <0.7 at the SEU was high. Children who were examined in the U8 and U9 had a better chance for a good visual acuity in the school entry examination.

Ziel einer Augenvorsorgeuntersuchung im Kindesalter ist das frühzeitige Erkennen von visuellen Störungen. Wichtig ist insbesondere das rechtzeitige Erkennen einer Amblyopie. Diese Schwachsichtigkeit entsteht im frühen Kindesalter und bedarf einer zeitnahen Behandlung. Ihre Prävalenz liegt in Deutschland bei 5,6 % im Alter von 35 bis 44 Jahren [5]; im europäischen Vergleich ist dies hoch: In Schweden liegt sie bei 1,1 % [13], in Dänemark bei 1,4 % [8]. In beiden Ländern sind Augenvorsorgeuntersuchungen im Vorschulalter etabliert.

Auch seltenere Augenerkrankungen wie eine kindliche Katarakt (Prävalenz 1:10.000) oder ein kongenitales Glaukom (Inzidenz 1:18.500) müssen möglichst früh diagnostiziert und behandelt werden, um eine optimale Sehentwicklung zu gewährleisten [10, 11, 14, 17]. Eine *augen*ärztliche Vorsorgeuntersuchung für Kinder ist derzeit nicht obligater Bestandteil des Leistungskataloges der gesetzlichen Krankenkassen. Im Jahr 2007 hat das Institut für Qualität und Wirtschaftlichkeit im Gesundheitswesen (IQWIG) festgestellt, dass derzeit weder ein Beleg noch ein Hinweis für den Nutzen einer augenärztlichen Vorsorgeuntersuchung im Kindesalter vorliege [2], bestätigt in einem *rapid report* 2015 [1]. Sehtests finden stattdessen im Rahmen der vom Kinder- und Jugendarzt bzw. Hausarzt durchgeführten Kindervorsorgeuntersuchungen U7a (Ende 3. Lebensjahr), U8 (Ende 4. Lebensjahr) und U9 (Beginn 6. Lebensjahr) statt; eine Inspektion der Augen ist bei allen Kindervorsorgeuntersuchungen vorgesehen. In Rheinland-Pfalz besteht, wie auch in anderen Bundesländern, ein verbindliches Einlade- und Meldewesen für Kindervorsorgeuntersuchungen: die Erziehungsberechtigten erhalten zunächst eine schriftliche Einladung zur jeweiligen Vorsorgeuntersuchung. Bleibt auch nach schriftlicher Erinnerung die Rückmeldung über eine Teilnahme aus, erfolgt eine Meldung an das betreffende Jugendamt.

Die Schuleingangsuntersuchung (SEU) ist in Rheinland-Pfalz und in anderen Bundesländern eine Pflichtuntersuchung, die im Jahr vor der Einschulung vom kinder- und jugendärztlichen Dienst der Gesundheitsämter durchgeführt wird; sie enthält einen Sehtest.

Nur wenige Studien beschäftigen sich mit der Effektivität von Augenvorsorgeuntersuchungen im Kindesalter [9]. In der vorliegenden Studie wurde untersucht, welcher Zusammenhang zwischen einer Teilnahme an den kinderärztlichen Vorsorgeuntersuchungen U8/U9 und der bei der Schuleingangsuntersuchung (SEU) gemessenen Sehschärfe besteht.

## Methoden

Basis dieser Querschnittstudie sind Daten der SEU in Rheinland-Pfalz. Diese ist gesetzlich vorgeschrieben und wird in 24 Landkreisen und 12 kreisfreien Städten durchgeführt. Sie erfolgt durch den kinder- und jugendärztlichen Dienst der Gesundheitsämter und findet zwischen Herbst und Sommer vor der Einschulung statt. Neben der Erhebung demografischer und gesundheitlicher Faktoren über einen Elternfragebogen erfolgen eine Überprüfung des Impfstatus, eine Dokumentation der in Anspruch genommenen Vorsorgeuntersuchungen, eine Anamneseerhebung, eine körperliche Untersuchung sowie ein Hör- und Sehtest.

Da der Landkreis Altenkirchen nicht einheitlich dokumentiert wurde, fehlen dessen Daten in der vorliegenden Studie. Die Daten der Einschulungsjahrgänge 2009/2010 bis 2014/2015 wurden in vollanonymisierter Form vom Ministerium für Soziales, Arbeit, Gesundheit und Demografie (MSAGD) des Landes Rheinland-Pfalz zur Verfügung gestellt.

Der Elternfragebogen umfasst – unter anderem – Angaben zum höchsten Schulabschluss beider Eltern, zu Hause gesprochene Sprachen, Tragen einer Brille und Augenarztbesuch in den zurückliegenden 12 Monaten. Anhand des Kinderuntersuchungsheftes werden die durchgeführten kinderärztlichen Vorsorgeuntersuchungen dokumentiert.

Der Sehtest wird mit Brille an einem Rodenstock-Sehtestgerät (R11 oder R21; Sehzeichen: Testscheibe 120 E‑Haken) monokular für Ferne und Nähe durchgeführt. Dokumentiert werden Sehschärfeangaben <1,0 (aber ≥0,7) und <0,7, da bei einer Sehschärfe <0,7 die Empfehlung zur augenärztlichen Kontrolle ausgesprochen wurde, sowie Auffälligkeiten beim räumlichen Sehen (Lang-Tests oder DeKa-Test [beide OCULUS Optikgräte GmbH, Wetzlar, Deutschland]) und Farbensehen.

### Statistische Analysen

Alle Kinder mit im Rahmen der SEU durchgeführtem Sehtest wurden in die vorliegende explorative Analyse eingeschlossen. Absolute und relative Häufigkeiten wurden für Sehschärfen <0,7 (ein- oder beidseitig jeweils mit Empfehlung zur augenärztlichen Kontrolle) für die gesamte Stichprobe, nach Einschulungsjahrgang und durchgeführter Vorsorgeuntersuchung stratifiziert berechnet.

Zur Analyse von Zusammenhängen zwischen der Teilnahme an Vorsorgeuntersuchungen und dem Vorliegen einer Sehschärfe <0,7 wurden Odds Ratios (OR) und deren 95 %-Konfidenzintervalle (95 %-KI) mittels multipler logistischer Regressionsanalyse berechnet. Es wurden 2 Modelle entwickelt: Modell 1 analysierte eine ein- oder beidseitig, Modell 2 eine beidseitig reduzierte Sehschärfe.

Potenzielle Confounder wurden zuvor mittels kausalem Diagramm („Directed Acyclic Graph“) bestimmt. Für die Teilnahme an der U8/U9 (gruppiert in Teilnahme an beiden „U“‑Untersuchungen, Teilnahme nur an U8, Teilnahme nur an U9, keine Teilnahme an beiden „U“‑Untersuchungen) wurde für Geschlecht, Alter (in Monaten), höchste Schulbildung der Eltern, Haushaltssprache und getragene Brille in den zurückliegenden 12 Monaten adjustiert. Bezüglich des Einflusses des Migrationshintergrundes als möglichem weiterem Confounder wurde eine Sensitivitätsanalyse durchgeführt. Die *p*-Werte werden im Rahmen dieser explorativen Analyse berichtet und haben deskriptiven Charakter. Ein Signifikanzniveau wurde nicht definiert. Alle Analysen wurden mit dem Softwarepaket R (Version 0.99.896) durchgeführt.

## Ergebnisse

Von 204.123 untersuchten Kindern konnten 165.316 Kinder (81,0 %; 79.232 Mädchen) in die Analysen eingeschlossen werden (Tab. 1; demografische Details). Ausgeschlossen werden mussten 13.152 (6,4 %) Kinder, welche kein Vorsorgebuch hatten, 1267 (0,6 %) Kinder mit falsch codierter augenärztlicher Prüfung und 24.388 (11,9 %) Kindern mit fehlenden Daten zur Sehschärfe.

**Table 1:** 

Jahrgang	2009/2010	2010/2011	2011/2012	2012/2013	2013/2014	2014/2015	Insgesamt
*N*	28.454	27.687	27.597	26.955	27.587	27.036	165.316
Weiblich (%)	13.584 (47,7)	13.165 (47,5)	13.370 (48,4)	12.855 (47,7)	13.256 (48,1)	13.002 (48,1)	79.232 (47,9)
Mittleres Alter (Jahre) bei SEU	5,8 ± 0,4	5,8 ± 0,4	5,8 ± 0,3	5,8 ± 0,4	5,8 ± 0,4	5,8 ± 0,4	5,8 ± 0,4
Anteil (%) SEU Visus <0,7 (einseitig)	2,39	2,35	2,29	2,23	2,56	2,94	2,46
Anteil (%) SEU Visus <0,7 (beidseits)	2,79	2,57	2,23	2,35	2,82	2,99	2,63
Teilnahmequote U8 (%)	88,01	88,27	94,84	97,55	97,83	97,97	94,02
Teilnahmequote U9 (%)	85,25	92,32	95,39	95,87	95,91	95,68	93,34
Anteil (%) der Kinder mit Teilnahme an allen Vorsorgeuntersuchungen (U1–U9 ohne U7a)	74,35	78,09	82,87	85,81	87,75	89,93	83,05

*SEU*  Schuleingangsuntersuchung, *U7a* kinderärztliche Vorsorgeuntersuchung am Ende des dritten Lebensjahres, *U8* kinderärztliche Vorsorgeuntersuchung am Ende des vierten Lebensjahres, *U9* kinderärztliche Vorsorgeuntersuchung zu Beginn des sechsten Lebensjahres

Es trugen 11.250 Kinder (6,8 %) eine Brille, 42.883 Kinder (25,9 %) waren vor der SEU zumindest einmal bei einem Augenarzt gewesen. Bei 149.381 Kindern (90,4 %) war die zu Hause gesprochene Sprache Deutsch.

Eine Sehschärfe <0,7 wurde bei 8416 Kindern (5,1 %) ermittelt. Im Beobachtungszeitraum von 2009 bis 2015 war dieser Anteil im Schuljahr 2014/2015 mit 5,9 % am höchsten (Tab. 1 und 2). Bei 4345 Kindern (2,6 %) war die Sehschärfe beider Augen niedrig, bei 4071 Kindern (2,5 %) nur die eines Auges. Der Anteil der Kinder, die an allen empfohlenen Vorsorgeuntersuchungen teilgenommen hatten, nahm im Beobachtungszeitraum zu (Tab. 1).

**Table 2:** 

	U8	U9
*Anzahl Teilnehmer*	155.424	154.312
Anteil (%) Kinder mit einseitig schlechter Sehschärfe	2,41	2,42
Anteil (%) Kinder mit beidseitig schlechter Sehschärfe	2,54	2,51
*Anzahl Nichtteilnehmer*	9892	11.004
Anteil (%) Kinder mit einseitig schlechter Sehschärfe	3,30	3,04
Anteil (%) Kinder mit beidseitig schlechter Sehschärfe	4,03	4,24

*U8* kinderärztliche Vorsorgeuntersuchung am Ende des vierten Lebensjahres, *U9* kinderärztliche Vorsorgeuntersuchung zu Beginn des sechsten Lebensjahres

### U8-Vorsorgeuntersuchung

Es hatten 155.424 Kinder (94,0 %) an der *U8* (Tab. 1) teilgenommen. Bei 2,4 % (*N* = 3745) dieser Kinder lag einseitig die Sehschärfe <0,7 (Tab. 2). Von diesen waren 37,6 % bei einem Augenarzt gewesen (*n* = 1408). Der Anteil von Kindern mit einer einseitig schlechten Sehschärfe lag bei Kindern ohne U8 bei 3,3 % (*n* = 326; zuvor stattgehabter Augenarztbesuch bei 21,2 %).

Bei 2,5 % (*N* = 3946) der U8-Teilnehmer wurde an beiden Augen eine Sehschärfe <0,7 gemessen (Augenarztbesuch bei 38,1 %). Eine beidseits schlechte Sehschärfe war bei 4,0 % (*N* = 399) der Kinder ohne U8 gemessen worden (vorheriger Augenarztbesuch fand bei 21,3 % statt).

Es fand sich ein klarer Zusammenhang zwischen der Teilnahme an der U8 und einem einseitigen SEU-Visus <0,7 (adjustiertes OR: 0,72; 95 %-KI: 0,62–0,84; *p* < 0,01; *N* = 121.356) als auch einem beidseitigen SEU-Visus <0,7 (adjustierte OR: 0,62; 95 %-KI: 0,54–0,71; *p* < 0,01; *N* = 121.496) (Tab. 3).

**Table 3:** 

Unabhängige Variablen	Modell einseitiger SEU-Visus <0,7	Modell beidseitiger SEU-Visus <0,7
U8 ohne U9	0,69 (0,52; 0,93)	0,74 (0,58; 0,95)
U9 ohne U8	0,76 (0,57; 1,03)	0,63 (0,49; 0,82)
U8 und U9	0,60 (0,47; 0,77)	0,45 (0,37; 0,55)
Weder U8 noch U9	Referenz	Referenz
Geschlecht (Weiblich)	1,07 (0,99; 1,15)	1,16 (1,08; 1,25)
Alter (Jahr)	0,84 (0,76; 0,93)	0,83 (0,75; 0,92)
Brille (ja)	3,08 (2,76; 3,44)	3,24 (2,92; 3,61)
*Höchster Bildungsabschluss* (Referenz Gymnasialabschluss oder Ähnliches)		
Realschulabschluss	1,18 (1,08; 1,28)	1,21 (1,11; 1,32)
Kein Schulabschluss oder Hauptschulabschluss	1,65 (1,50; 1,82)	1,81 (1,65; 1,99)
Augenarztbesuch (ja)	1,26 (1,16; 1,38)	1,32 (1,22; 1,44)
Gesprochene Sprache (kein Deutsch)	1,17 (0,96; 1,40)	1,45 (1,22; 1,71)

*U8* kinderärztliche Vorsorgeuntersuchung am Ende des vierten Lebensjahres, *U9* kinderärztliche Vorsorgeuntersuchung zu Beginn des sechsten Lebensjahres

### U9-Vorsorgeuntersuchung

Es hatten 154.312 Kinder (93,34 %) an der *U9* (Tab. 1) teilgenommen. Bei 2,4 % (*N* = 3736) dieser Kinder wurde einseitig eine Sehschärfe <0,7 gemessen (Tab. 2), 3734 (37,47 %) waren bei einem Augenarzt gewesen. Der Anteil von Kindern mit einer einseitig schlechten Sehschärfe lag bei Kindern ohne U9 (*N* = 11.004) bei 3,04 % (*N* = 335), bei 23,28 % (*N* = 78) war ein Augenarztbesuch dokumentiert.

Bei 2,51 % (*N* = 3878) der U9-Teilnehmer wurde an beiden Augen eine Sehschärfe <0,7 gemessen, 38,11 % hatten im Vorfeld einen Augenarzt aufgesucht. Eine beidseits schlechte Sehschärfe wurde bei 4,24 % (*N* = 467) der Kinder ohne U9 gemessen, bei 23,55 % war ein Augenarztbesuch angegeben.

Es fand sich ein klarer Zusammenhang zwischen der Teilnahme an der U9 und einem einseitigen SEU-Visus <0,7 (adjustierte OR: 0,79; 95 %-KI: 0,68–0,91; *p* < 0,01; *N* = 121.356) sowie für beidseitigen SEU-Visus <0,7 (adjustierte OR: 0,56; 95 %-KI: 0,49–0,64; *p* < 0,01; *N* = 121.496) (Tab. 3).

### U8/U9-Wechselwirkungen

Es hatten 147.856 (89,4 %) Kinder sowohl an U8 als auch an U9 teilgenommen; 6456 (3,9 %) hatten zwar an der U9, nicht aber an U8, 7568 (4,6 %) Kinder an der U8, nicht aber an der U9 und 3436 (2,1 %) Kinder weder an U8 noch an U9 teilgenommen.

Teilnahme an der U8 und Teilnahme an der U9 senkte die Chance für einen einseitigen SEU-Visus < 0,7 (adjustierte OR 0,60; 95 %-KI: 0,47–0,77; *p* < 0,01) und einen beidseitigen SEU-Visus < 0,7 (adjustierte OR: 0,45; 95 %-KI: 0,37–0,55; *p* < 0,01). Eine Teilnahme nur an U8 und nicht an U9 senkte die Chance für einen einseitigen SEU-Visus < 0,7 (adjustierte OR 0,69; 95 %-KI: 0,52–0,93; *p* = 0,013) und einen beidseitigen SEU-Visus <0,7 (adjustierte OR: 0,74; 95 %-KI: 0,58–0,95; *p* = 0,016). Wurde nur an der U9 und nicht an der U8 teilgenommen, nahm die Chance für einen einseitigen SEU-Visus < 0,7 (adjustierte OR: 0,76; 95 %-KI: 0,57–1,03; *p* = 0,074) und einen beidseitigen SEU-Visus < 0,7 (adjustierte OR: 0,63; 95 %-KI: 0,49–0,82; *p* < 0,01) ebenso ab.

## Diskussion

Der Anteil von Kindern mit reduzierter Sehschärfe vor der Einschulung betrug in der vorliegenden Studie unter allen 165.316 untersuchten Kindern 5,1 %. Eine Teilnahme an U8/U9 (im Rahmen derer ein Sehtest erfolgt) war mit einer Reduktion des Anteils von ein- oder beidseitiger SEU-Sehschärfe <0,7 assoziiert: Für einen einseitigen SEU-Visus <0,7 betrug die adjustierte OR 0,72 nach Teilnahme an U8 und OR 0,79 nach Teilnahme an U9. Eine Teilnahme an U8 oder U9 verringerte also die Wahrscheinlichkeit für einen reduzierten Visus bei der SEU um etwa ein Viertel. Fand hingegen jeweils eine dieser beiden Voruntersuchungen nicht statt, erhöhte dies die Wahrscheinlichkeit für einen reduzierten Visus bei der SEU um etwa ein Viertel. Bei fehlender Teilnahme sowohl an U8 und U9 war diese Wahrscheinlichkeit fast doppelt so hoch.

Beobachtungsstudien wie die Vorliegende unterliegen der Gefahr von Bias, insbesondere durch das Fehlen geeigneter Kontrollen und dem residualen Confounding, also dem Nichtbeachten verzerrender Störgrößen. U8 und U9 waren zum Zeitpunkt des Beginns der Studie (2009) bereits seit Jahrzehnten etabliert mit jeweils hohen Teilnahmequoten. Nichtteilnehmer an Kindervorsorgeuntersuchungen stammen insbesondere aus Familien mit Migrationshintergrund sowie sozial schwachen Familien [15]. Dies wurde zwar in der Analyse berücksichtigt, allerdings können sich nicht teilnehmende Kinder und ihre Familien auch hinsichtlich anderer relevanter, aber nicht berücksichtigter Faktoren unterscheiden, wie z. B. Freizeitverhalten, Medienkonsum, soziale Vernachlässigung oder chronische Erkrankung. Letztere gehen mit einem höheren Risiko für Amblyopie einher [18]. Die Ergebnisse zur U8 und zur U9 sind daher kein Nachweis einer Effektivität dieser Untersuchungen hinsichtlich der Augen; stattdessen erlauben sie die Vermutung, dass Risikogruppen durch das Meldesystem der Kindervorsorgeuntersuchungen der Jahre 2007 bis 2012 nicht vollständig erfasst wurden, erkennbar am hohen Anteil von Kindern mit reduzierter Sehschärfe kurz vor der Einschulung. Die Kindervorsorgeuntersuchung U7a hingegen, die am Ende des 3. Lebensjahres stattfindet, reduziert den Anteil bei der SEU schlecht sehender Kinder nicht [6]. Sie wurde erst 2008 in den Leistungskatalog der gesetzlichen Krankenversicherung aufgenommen, sodass ähnlich einem natürlichen Experiment eine Beobachtung vor und nach Einführung möglich war. Teilnehmer und Nichtteilnehmer sind daher strukturähnlicher als bei U8 und U9.

Im Verlauf des Einschulungsjahrgangs 2009/2010 bis 2014/2015 war der Anteil von Kindern mit reduziertem SEU-Visus im Einschulungsjahrgang 2014/2015 mit 5,17 % am höchsten (Tab. 1). Zeitgleich stiegen paradoxerweise die Teilnehmerquoten an den kinderärztlichen Vorsorgeuntersuchungen – Hauptursache dafür ist vermutlich das zwischenzeitlich eingeführte, verbindliche Einlade- und Meldewesen für die Kindervorsorgeuntersuchungen; überdies wurde 2008 mit der U7a eine weitere kinderärztliche Vorsorgeuntersuchung eingeführt, die einen Sehschärfentest beinhaltet.

Bei rund der Hälfte der Kinder mit reduzierter Sehschärfe wurde an lediglich einem Auge ein Visus <0,7 gemessen. Kinder mit einseitig reduzierter Sehschärfe sind im Alltag typischerweise unauffällig, weil die einseitig schlechte Sehschärfe mit dem anderen Auge kompensiert werden kann. Dies unterstreicht die Wichtigkeit des korrekt durchgeführten Sehschärfentests: Die Sehschärfe muss monokular geprüft werden. Gelingt dies nicht, muss der Sehtest als auffällig eingestuft und kurzfristig zumindest wiederholt werden. Wichtig ist in diesem Zusammenhang auch die Verwendung eines geeigneten Sehtests, z. B. LEA-Symbole, E‑Haken oder Landolt-Ringe.

Obschon ein Vergleich mit Studien aus anderen europäischen Ländern insbesondere wegen unterschiedlicher Untersuchungsmethoden und -alter der untersuchten Kinder schwierig ist, scheint der Anteil schlecht sehender Vorschulkinder in Deutschland hoch zu sein: In den Niederlanden fanden sich in einer Kohorte von 7‑jährigen Kindern lediglich 1,6 % mit einer Sehschärfe ≥0,2 logMAR (≦0,63 Snellen-Visus) [4]. Eine naheliegende Erklärung dafür ist, dass bei niederländischen Kindern in den ersten 2 Lebensjahren 5 Augenuntersuchungen stattfinden. Möglicherweise reduziert auch eine bessere Compliance aufgrund des etwas höheren Alters bei der Sehschärfenuntersuchung der niederländischen Kinder den Anteil schlecht sehender Kinder. Eine britische Studie testete die monokulare Sehschärfe 7,5-jähriger Kinder: Nach einem Sehscreening im Alter von 4 bis 5 Jahren lag der Anteil von Kindern mit einem Visus <6/9 (<0,67) bei 1,9 %. War dieses Screening nicht erfolgt, betrug der Anteil 3,4 % [19]. Für das Visuskriterium 0,5 (0,3 logMAR) lagen die Anteile bei 0,7 % (mit Screening) und 1,3 % (ohne Screening). Ein intensives orthoptisches Screening in den ersten 37 Lebensmonaten zeigte in Großbritannien einen deutlichen Effekt auf die Amblyopieprävalenz im Alter von 7,5 Jahren: Nach orthoptischen Vorsorgeuntersuchungen im Alter von 8, 12, 18, 25, 31 und 37 Monaten betrug diese 0,6 %. Fand lediglich eine orthoptische Untersuchung im Alter von 37 Monaten statt, lag der Amblyopieanteil bei 1,8 %. Eines der Kriterien für Amblyopie war eine Sehschärfe <0,5 (größer 0,3 logMAR) am besseren Auge, was den Vergleich mit der vorliegenden Studie, bei der das Visuskriterium <0,7 war, erschwert. Darüber hinaus haben die SEU nicht den Anspruch, Amblyopien zu entdecken. Zusammengefasst lässt sich feststellen, dass diese europäischen Daten ebenso wie die vorliegende Studie auf einen positiven Effekt eines frühen Augenscreenings hinweisen. Andererseits hat die in Deutschland im Jahr 2006 neu eingeführte Kindervorsorgeuntersuchung U7a am Ende des 3. Lebensjahres keinen Einfluss auf den Anteil schlecht sehender Kinder bei der SEU [6]. Zu ähnlichen Ergebnissen kam 2004 die „Tübinger Kindergartenstudie“ [3], die feststellte, dass die Teilnahme an Kindervorsorgeuntersuchungen keinen Einfluss auf die Erkennung einer Amblyopie hat. Berücksichtigt man zudem den hohen Anteil schlecht sehender Vorschulkinder, muss dies Anlass geben, die bislang durchgeführten Augenvorsorgeuntersuchungen zu überdenken und zu optimieren. Insbesondere müssen standarisierte Untersuchungsabläufe und -inhalte eingeführt werden.

Für den hohen Anteil von Kindern (2,3 %) mit beidseits SEU-Visus <0,7 sind mehrere Ursachen denkbar. Eine beidseitige Visusminderung kann durch höhere symmetrische Ametropien begründet sein, die Prävalenz bei Kindern in diesem Alter liegt bei rund 30 % [7, 12, 16]. Eine noch nicht ausreichende Konzentrations- und Mitarbeitsfähigkeit der Kinder kann ebenfalls ein Grund sein: Im augenärztlichen Alltag sind Kinder mit schlechtem SEU-Visus, die bei der Kontrolluntersuchung doch eine regelrechte Sehschärfe aufweisen, keine Seltenheit. Sehschärfenrelevante Augenpathologien hingegen, wie z. B. eine beidseitige kindliche Katarakt oder eine sich früh manifestierende Netzhautdystrophie haben niedrige Prävalenzen, für Katarakt liegt sie bei etwa 0,03 % [20].

### Einschränkungen

Den vorliegenden Daten können keinesfalls die Ursachen für die reduzierte SEU-Sehschärfe entnommen werden; der Anteil schlecht sehender Kinder wird durch mangelnde Compliance bzw. Konzentrationsfähigkeit möglicherweise überschätzt. Andererseits wäre auch eine Unterschätzung dieses Anteils denkbar: Die Sehschärfe wird bei der SEU mit Einzeloptotypen gemessen, die für viele Amblyopien typischen Trennschwierigkeiten bei eng stehenden Reihenoptotypen können übersehen werden. Zudem stellt die SEU-Sehschärfenmessung keine augenärztliche Sehschärfenprüfung mit optimaler Refraktion dar, sondern eine Screeninguntersuchung durch Ärzte des öffentlichen Gesundheitsdienstes. Eine Heterogenität in der Erhebung in verschiedenen Landkreisen ist möglich, auch wenn standardisierte Vorgehensbeschreibungen diese bestmöglich definieren.

## Fazit

- Die Prävalenz von Kindern mit im Rahmen der SEU reduzierter Sehschärfe ist im europäischen Vergleich hoch.
- Hoch ist insbesondere der Anteil von Kindern, die bei der SEU mit beiden Augen schlecht gesehen haben.
- Sowohl eine Nichtteilnahme an U8 als auch an U9 erhöhen das Risiko für eine reduzierte SEU-Sehschärfe um rund ein Drittel, eine Nichtteilnahme an beiden Vorsorgeuntersuchungen verdoppelt es.
- Gut ein Viertel der künftigen Schulkinder war vor der SEU zumindest einmal beim Augenarzt gewesen.
